# Supplementary material for: Attenuated Total Reflection Fourier-Transform Infrared Spectral Discrimination in Human Tissue of Oesophageal Transformation to Adenocarcinoma
Source: J Pers Med. 2023 Aug 20;13(8):1277. doi: 10.3390/jpm13081277 (PMC10455976; doi:10.3390/jpm13081277)
Supplement: Supplementary file 1 [file jpm-13-01277-s001.zip › jpm-2452690-supplementary.pdf]

Table S1. Category-distinguishing wavenumbers for Normal *vs.* Inflammatory *vs.* Barrett's *vs.* LGD *vs.* HGD *vs.* OAC using spectra tissues samples obtained for SPA-QDA model

Wavenumbers (cm^{-1})	Tentative Assignments
~1393	Symmetric CH_3 bending of the methyl groups of proteins [29, 41] or aliphatic side groups of the amino acid residues [30]
~1540	Protein amide II absorption – predominately b-sheet of amide II [28]
~1589	Ring C-C stretching of phenyl [42]
~1640	Amide I band [23]
~1690	Peak of nucleic acids due to the base carbonyl stretching and ring breathing mode [30]
~1745	Ester group (C=O) vibration of lipids [28, 31]

References

41 Wang, H.P., Wang, H.-C., Huang, Y.-J. Microscopic FTIR studies of lung cancer cells in pleural fluid. *Science of the Total Environment*, 1997, 204: 283–287.

29 Wood, B.R., Quinn, M.A., Burden, F.R., McNaughton, D. An investigation into FT-IR spectroscopy as a bio-diagnostic tool for cervical cancer. *Biospectroscopy*, 1996, 2: 143–153.

30 Chiriboga, L., Xie, P., Yee, H., Vigorita, V., Zarou, D., Zakim, D., Diem, M. Infrared spectroscopy of human tissue. I. Differentiation and maturation of epithelial cells in the human cervix. *Biospectroscopy*, 1998, 4: 47–53.

28 Wu, J.-G., Xu, Y.-Z., Sun, C.-W., Soloway, R.D., Xu, D.-F., Wu, Q.-G., Sun, K.-H., Weng, S.-F., and Xu, G.-X. Distinguishing malignant from normal oral tissues using FTIR fiber-optic techniques. *Biopolymer* (*Biospectroscopy*), 2001, 62: 185–192

42 Movasaghi, Z., Rehman, S., ur Rehman, I. Fourier Transform Infrared (FTIR) Spectroscopy of Biological Tissues. *Applied Spectroscopy Reviews*, 2008, 43: 134–179.

23 Wood, B.R., Quinn, M.A., Tait, B., Ashdown, M., Hislop, T., Romeo, M., McNaughton, D. FTIR microspectroscopic study of cell types and potential confounding variables in screening for cervical malignancies. *Biospectroscopy*, 1998, 4: 75–91.

31 Sukuta, S., Bruch, R. Factor analysis of cancer Fourier transform infrared evanescent wave fiberoptical (FTIR-FEW) spectra. *Lasers in Surgery and Medicine*, 1999, 24: 382–388.
